# Associations of neighborhood built, safety, and social environment with walking to and from school among elementary school-aged children in Chiba, Japan

**DOI:** 10.1186/s12966-021-01202-y

**Published:** 2021-11-27

**Authors:** Kimihiro Hino, Erika Ikeda, Saiko Sadahiro, Shigeru Inoue

**Affiliations:** 1 Department of Urban Engineering, Graduate School of Engineering, The University of Tokyo, 7-3-1 Hongo, Bunkyo-ku, Tokyo, 113-8656 Japan; 2 MRC Epidemiology Unit, School of Clinical Medicine, University of Cambridge, Box 285, Institute of Metabolic Science, Cambridge Biomedical Campus, Cambridge, CB2 0QQ UK; 3 Faculty of Education, Chiba University, 1-33 Yayoi-cho, Inage-ku, Chiba-shi, Chiba, 263-8522 Japan; 4 Department of Preventive Medicine and Public Health, Tokyo Medical University, 6-1-1 Shinjuku, Shinjuku-ku, Tokyo, 160-8402 Japan

**Keywords:** Active travel, Commuting, Safety, Crime prevention, New town

## Abstract

**Background:**

Although it is globally known that Japan has high prevalence of active school travel among children, there are few international studies on Japanese children’s school travel. Moreover, only few studies have focused on the differences in their mode of travel between to-school and from-school. This study examined the associations of neighborhood built, safety, and social environments with walking to/from school among elementary school-aged children in Chiba, Japan.

**Methods:**

We conducted an online survey with 1545 parents of children aged 6–12 years residing in Chiba between 25 and 27 November 2020 during the COVID-19 pandemic. A neighborhood was defined as the area of a postcode provided by the participants. Each neighborhood environment was assessed based on the built environment (new town designation, walkability, distance to school, population density), social environment (neighborhood cohesion and connection), and safety (CCTVs, a road section for walking alone, safety volunteers). Neighborhood walkability was measured using subscales of the Neighborhood Environment Walkability Scale (youth and abbreviated versions) including crime safety and traffic safety. Parents’ perceived influence of COVID-19 on school commuting and after-school activities were also included in the model as covariates. Walking to and from school were separately analyzed using multinomial logistic regressions, where new towns and walkability were computed separately as explanatory variables.

**Results:**

Four fifths of children walked to and from school daily. Walking to school was positively associated with crime safety, neighborhood connections, and schools sited in new towns. Walking from school had positive associations with traffic safety, neighborhood cohesion, and CCTVs, but negative associations with safety volunteers and after-school activities. The presence of a section for walking alone and perceived influence of COVID-19 had negative associations with walking to and from school.

**Conclusions:**

Recent social changes such as declining birthrate, decline in public elementary schools, and increasing after-school activities may change parental attitudes toward children’s walking to/from school, and subsequently, their mode of school travel over time. To maintain the high prevalence of walking to/from school in Japan, multidisciplinary approaches involving different stakeholders from education, public health, and urban planning are required to overcome sectionalism and support this behavior in the long term.

**Supplementary Information:**

The online version contains supplementary material available at 10.1186/s12966-021-01202-y.

## Background

Physical activity (PA) is recognized as a key determinant of physical, physiological, developmental, mental, cognitive, and social health among children. However, the prevalence of insufficient PA is high globally [1, 2]. Promotion of active school travel (e.g., walking and cycling to/from school) may be a way to improve children’s health due to its association with levels of PA [3–5]. In Japan, more than 90% of children travel to school on foot, which may partly contribute to the relatively low prevalence of childhood obesity and being overweight [6]. According to the 2018 Report Cards on Physical Activity for Children and Youth from 49 countries (also known as the Global Matrix 3.0) [7], Japan was highly rated as “A-” (i.e., 80–86% prevalence) for active transportation [8].

Previous research has shown that active school travel was positively associated with neighborhood physical and social environments, safety, walkability, and neighborhood social interactions, and negatively associated with travel distance and car ownership [9, 10]. In particular, safety has been identified as the core concept of school travel policies [11, 12]; most studies have focused on traffic safety [13–15], while few have focused on safety from crimes or stranger danger [16–18]. The low crime rate in Japan and well-established safety interventions could be the reasons for the high rates of active school travel. For example, school staff and local volunteers in Japan regularly supervise road crossings in places that have considerable traffic to ensure that children cross safely [19]. However, in recent years, there has been a high level of anxiety about crimes against children in Japan [20]. Physical offenses against children under the age of 13 (equivalent to elementary school children or younger) are more likely to occur during school travel hours on weekdays, especially when returning from school (around 3–6 pm.) [21]. In 2018, the national government formulated the “Crime Prevention Plan for Children Commuting to/from School” urging the police, schools, local residents, and local governments to improve the environment, such as intensive watching and CCTV installation on the road sections where children walk alone while travelling to and from school [22].

Another reason for high rates of active school travel may be that a large proportion of mothers stay at home compared with other high-income countries [19], which helps children return home straight from school. However, this trend has shifted dramatically over the past two decades [23], with more women and mothers being employed. Consequently, an increasing number of children are not able to return home directly from school, and the time for going home has diversified. These social changes have decreased the number of local volunteers for children’s safety, making it difficult to supervise children returning from school [22].

Despite these social phenomena in relation to active school travel in Japan, there are few international studies on Japanese children’s school travel. Moreover, only few studies have focused on the differences in their mode of travel between to-school and from-school. To fill this research gap, this study aimed to examine the associations between the neighborhood built, safety, and social environments of elementary school children and their walking to and from schools in Chiba prefecture, Japan. We explored these associations of neighborhood safety as a factor of walkability that has been pursued through urban planning and design.

## Methods

### Study design and setting

In this cross-sectional study, we conducted an online survey with 1545 parents of elementary school-aged children (6–12 years) who resided in Chiba prefecture, between 25 and 27 November 2020. At this time, a state of emergency due to COVID-19 had not been declared in Chiba prefecture, and therefore, the elementary schools were open (schools were temporarily closed due to COVID-19 only between March and May 2020). The online survey included questions on children’s modes of travel to and from school, neighborhood environments, and socio-demographic information. All participants provided informed consent prior to the commencement of the survey. This study was conducted in accordance with the STROBE guidelines for cross-sectional studies (Additional file 1).

We chose Chiba prefecture (population: 6.28 million, population density 1218 per hectare), which consists of 54 municipalities (37 cities, 16 towns, and one village), as the target study area because it has a mix of urban, suburban, and rural areas in a wide prefecture area [24]. Located on the outskirts of Tokyo, Chiba prefecture has several “new towns” (NT) built by municipalities and the Japan Housing Corporation to accommodate people who migrated to Tokyo during the postwar high-growth period from the late 1950s to the early 1970s. Following precedents in the UK, Japanese NTs were designed and built to ensure the safety and security of working-age families and their children [25]. One of their features is a web of greenways connecting abundant parks and greenspaces. These well-connected greenways are separated from the road network for traffic safety and are expected to function as corridors for pedestrian and bicycle commuters [26]. We hypothesized that the built environmental features of NTs might be associated with children’s school travel modes.

Each of the 54 municipalities has a municipal board of education (local educational authority) in charge of the public schools [27]. It is a common practice for each municipal board of education to establish walking to school as compulsory if the school is located within a certain distance (approximately 2 km) of the student’s home [19]. However, in recent years, elementary schools have been integrated and the school catchment area has widened due to the declining birthrate, which may in turn have led to less walking to/from school [28].

### Participants

Participants were parents of elementary school-aged children who lived in Chiba prefecture. An invitation email that contained an outline of the study and a link to the online survey was sent to research panelists who lived in the prefecture and voluntarily registered with a third-party research company. Of the 90,050 research panelists, those who passed a screening question of having elementary school-aged children proceeded to the main survey questions. The survey link was deactivated when reaching a pre-determined target of 1500 respondents.

### Measures

#### School travel modes

Participants were asked, “*In the past month, how often did your child use a mode of travel to and from school other than walking?*”, with five response options of ‘0 days,’ ‘1 or 2 days,’ 3–5 days,’ 6–10 days,’ and ‘11 days or more.’ The responses were recorded separately for ‘to’ and ‘from’ school. The school travel modes were recoded into three categories: everyday walkers (0 days), frequent walkers (more than half a month: 1 or 2 days, 3–5 days, 6–10 days), and less frequent walkers (less than half a month: 11 days or more).

#### Neighborhood environment

A neighborhood was defined as the area of a postcode (which is a proxy for home location) provided by the participants. The neighborhood environment for each participant was assessed based on the built environment, social environment, and neighborhood safety [18, 29].

##### Built environments

**Fig. 1:**
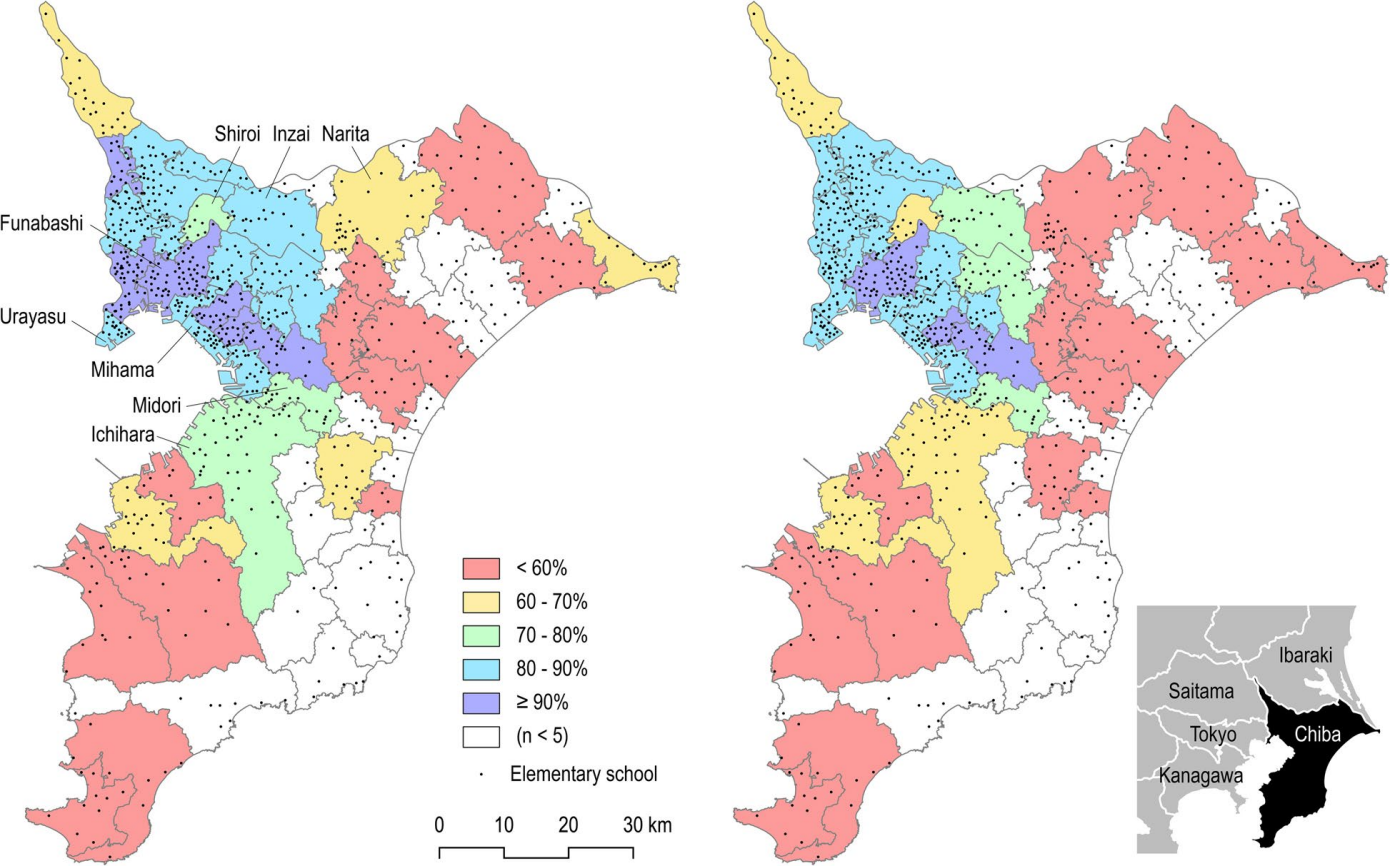
Prevalence of children walking to (left) and from (right) school every day by municipality in Chiba prefecture. n = number of participants

Neighborhood built environments were determined based on four measures: NT, walkability, distance to school, and population density. NT was measured to determine whether the nearest elementary school from the participants’ homes was located in NTs. The NTs include the Chiba NT (1930 ha; in Funabashi City, Inzai City, and Shiroi City), Ichihara NT (974 ha; in Chiba City and Ichihara City), Urayasu (1241 ha; in Urayasu city), Kaihin NT (1293 ha; in Chiba city), and Narita NT (482 ha; in Narita city), which are the five major NTs in Chiba prefecture (see Fig. 1) [30]. Neighborhood walkability was measured using four subscales of the Neighborhood Environment Walkability Scale in Youth (NEWS-Y) [31] and one item from the Abbreviated Neighborhood Environment Walkability Scale (ANEWS) [32]. The NEWS-Y subscales included walking facilities (three items), aesthetics (four items), traffic safety (seven items), and crime safety (six items). The ANEWS item was derived from the walking facility (“sidewalks are separated from the road/traffic in our neighborhood by guardrails and curbs”). Response scales (1 = agree, 2 = somewhat agree, 3 = neither agree nor disagree, 4 = somewhat disagree, 5 = disagree) were reverse-coded where appropriate, and the mean scores of each subscale were calculated. The shortest distance from a participant’s neighborhood (i.e., gravity center) to the nearest elementary school was calculated using ArcGIS 10.8 (Esri Japan Corporation, Tokyo, Japan). Information on school locations was obtained using the Zenkoku Gakko Data [33]. Neighborhood population density, obtained from Chiba prefecture [34], was used as a proxy for the availability of commercial facilities and public transport [35].

##### Social environments

Neighborhood social environments were assessed based on neighborhood cohesion (i.e., to what extent participants trusted their neighbors; nine items) and neighborhood connection (i.e., to what extent participants connected to their neighbors; five items) and was rated on a 5-point Likert scale (1 = agree, 2 = somewhat agree, 3 = neither agree nor disagree, 4 = somewhat disagree, and 5 = disagree) [18, 36]. The scales were reverse-coded where appropriate, and the mean scores of both subscales were calculated.

##### Safety environment

Neighborhood safety on children’s travel routes to and from school was assessed by (1) whether there were any closed-circuit televisions (CCTVs); (2) whether there was a road section where children walked from school more than 100 m alone; and (3) whether parents and/or local residents voluntarily watched out for children on streets to/from school on a daily basis. Response options were ‘yes,’ ‘no,’ and ‘not sure.’ One general response to the CCTV item was recorded per participant, which applied to both the ‘TO’ and ‘FROM’ school models (see the Statistical Analysis section for further details). Walks over 100 m alone were specifically asked on return from school, but its response was also used for the TO school model given that both situations were likely to be similar. Different responses between to and from schools were recorded for the safety volunteer item.

#### Parent-perceived influence of COVID-19 on school commuting

At the time of the survey (25–27 November 2020), the number of people infected with coronavirus disease 2019 (COVID-19) in Japan exceeded 2500 per day. In response to COVID-19 and the related physical distancing advice, it was assumed that children might have changed their school travel behavior. Therefore, we asked whether children’s modes of travel to and from school had been influenced by COVID-19, with response options of ‘yes,’ ‘no,’ and ‘not sure’.

#### After-school activity

Modes of travel from school can be diversified because more children participate in after-school activities. The frequency of going directly from school to public facilities for children (‘*Jidou-kan*’ where children spend time playing and studying) or after school lessons and clubs outside school was measured, and responses ranged from to 0–5 days per weekday.

#### Socio-demographic characteristics

Children’s school grades (1st–6th grade; ages 6–12 years), sex (male or female), and the number of cars owned by the household (1–5, 6, or more) were assessed [18, 37, 38].

### Statistical analysis

Descriptive statistics, which included numbers and percentages, were calculated for all variables. For continuous variables, t-tests were used to test the differences in the means between children who attended schools in NTs and those who did not. Associations between neighborhood environments (built environment, social environment, and safety) and children’s school travel modes were examined using multinomial logistic regression analysis because of their non-linear relationships [39, 40]. In each outcome model (TO and FROM school), NT and neighborhood walkability were computed separately as explanatory variables due to a collinearity issue between these variables. Thus, four models were tested: TO school (1) NT model, (2) neighborhood walkability model, FROM school (3) NT model, and (4) neighborhood walkability model. Distance to school, population density, parent-perceived influence of COVID-19, after-school activities, and socio-demographic characteristics were used as control variables. Pseudo R-squared for the multinomial logistic regression model (Cox & Snell, Nagelkerke, and McFadden) was calculated to assess the model performance. The significance level was set at *P* < 0.05. All statistical analyses were conducted using IBM SPSS Statistics 26 (IBM Corp., Armonk, NY, USA).

## Results

A total of 1545 participants completed the online survey. After excluding 40 children who attended private elementary schools and eight children who lived outside the prefecture, data from 1497 participants were included in the analyses. Table 1 shows the descriptive statistics of all the variables. Most children walked to (82.0%) and from (79.9%) schools every day. Only 6.1% (to school) and 7.8% (from school) children used travel modes other than walking for 10 days or more per month. In total, more children walked to school than from school. When we compared the percentage of “everyday walkers” by geographical region, a higher percentage was observed in the western municipalities near Tokyo (Fig. 1).

**Table 1:** Descriptive statistics of responses

**Categorical variables**	**Category**	**n**	**%**
**Socio-demographic characteristics:**			
Child school grade	Grade 1	290	19.4%
	Grade 2	292	19.5%
	Grade 3	244	16.3%
	Grade 4	230	15.4%
	Grade 5	229	15.3%
	Grade 6	212	14.2%
Child’s sex	Boy	765	51.1%
	Girl	732	48.9%
Number of household cars owned	0	141	9.4%
	1	1005	67.1%
	≥ 2	351	23.4%
**School travel mode:**			
Number of days NOT walking per month (to school)	0(“Everyday walkers”)	1227	82.0%
	1–2(“Frequent walkers”)	102	6.8%
	3–5(“Frequent walkers”)	57	3.8%
	6–10(“Frequent walkers”)	19	1.3%
	> 10(“Less frequent walkers”)	92	6.1%
Number of days NOT walking per month (from school)	0(“Everyday walkers”)	1196	79.9%
	1–2(“Frequent walkers”)	80	5.3%
	3–5(“Frequent walkers”)	73	4.9%
	6–10(“Frequent walkers”)	31	2.1%
	> 10(“Less frequent walkers”)	117	7.8%
**Safety:**			
Safety volunteer (to school)	Yes	1202	80.3%
	No	199	13.3%
	Not sure	96	6.4%
Safety volunteer (from school)	Yes	808	54.0%
	No	487	32.5%
	Not sure	202	13.5%
CCTV on school way	Yes	290	19.4%
	No	689	46.0%
	Not sure	518	34.6%
Walking over 100 m alone	Yes	737	49.2%
	No	657	43.9%
	Not sure	103	6.9%
**Parent-perceived influence of COVID-19 on school commuting:**	Yes	95	6.3%
	No	1374	91.8%
	Not sure	28	1.9%
**After-school activity (days):**	0	1151	76.9%
	1	133	8.9%
	2	103	6.9%
	3	51	3.4%
	4	26	1.7%
	5	33	2.2%
**Continuous variables**		**Mean**	**SD**
**Built environments**^**a**^**:**			
Neighborhood walkability			
Walking facilities		2.35	0.72
Aesthetics		2.28	0.67
Traffic safety		2.46	0.40
Crime safety		2.68	0.57
Distance to school (m)		564.50	401.04
Population density (per km^2^)		103.82	79.56
**Social environments**^**a**^**:**			
Neighborhood cohesion		2.97	0.45
Neighborhood connection		3.17	0.79

CCTV, closed-circuit television; COVID-19, coronavirus disease 2019; SD, standard deviation

^a^Distance to school and population density were objectively measured, and the other built and social environment variables were self-reported

**Fig. 2:**
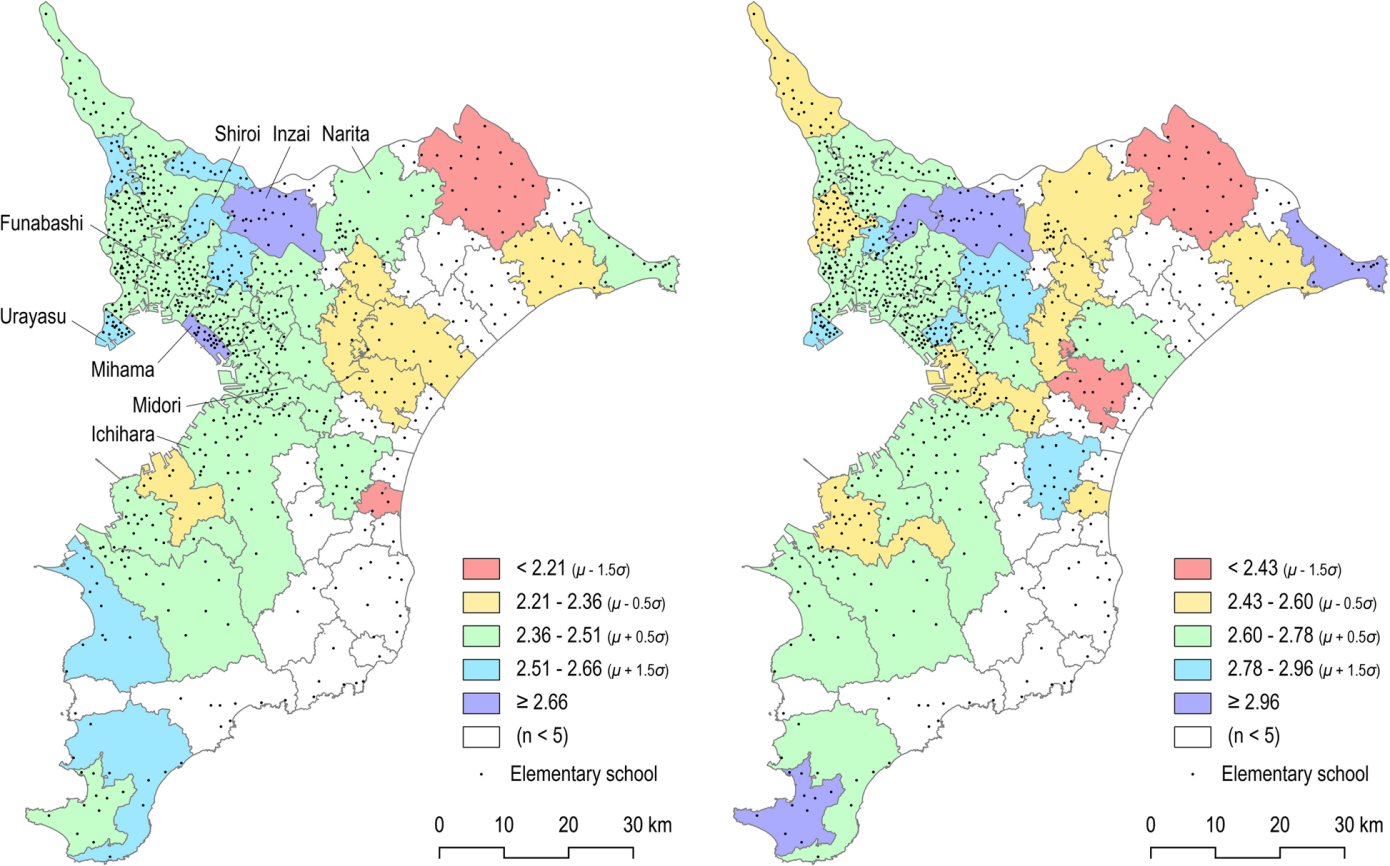
Mean scores of neighborhood crime (left) and traffic safety (right) by municipality in Chiba prefecture. n = number of participants; μ = mean of scores by municipality (the higher, the safer); σ = standard deviation of scores by municipality

Compared to the non-NT group, the NT group had significantly higher perceived neighborhood walkability scores but non-significantly lower social-environment scores. Distance to school was significantly shorter in the NT group than in the non-NT group (Table 2). When we compared neighborhood crime safety (i.e., a subscale of walkability), Inzai City and Shiroi City in which the Chiba NT was developed and areas on the peninsula had a higher mean score (safer) than the other regions (Fig. 2). Similarly, the level of neighborhood traffic safety (i.e., a subscale of walkability) was higher in the two abovementioned cities, Mihama Ward, in which the Kaihin NT was developed, and municipalities in the eastern part of the prefecture (Fig. 2).

**Table 2:** Mean and standard deviation of neighborhood environmental variables and their differences between new towns and non-new towns

	New towns	Non-new towns	*P*-value
n	152	1345	
**Built environments:**			
Neighborhood walkability			
Walking facilities	2.99 (0.60)	2.28 (0.69)	< 0.001
Aesthetics	2.87 (0.62)	2.22 (0.65)	< 0.001
Traffic safety	2.68 (0.36)	2.44 (0.39)	< 0.001
Crime safety	2.81 (0.60)	2.67 (0.57)	0.004
Distance to school (m)	433.52 (286.77)	579.3 (409.42)	< 0.001
Population density (per km^2^)	109.39 (59.56)	103.19 (81.51)	0.246
**Social environments:**			
Neighborhood cohesion	2.97 (0.43)	2.97 (0.45)	0.937
Neighborhood connection	3.11 (0.87)	3.18 (0.78)	0.337

### Associations of neighborhood environments with active school travel

Results from the multinomial logistic regression analyses of walking to and from school are shown in Tables 3 and 4, respectively. In all analyses, comparisons were made between the less frequent walkers (reference group) and the everyday walkers or frequent walkers. Additionally, the everyday walkers (reference group) were compared with the frequent walkers to examine the differences between the two groups. In Table 3 (walking ‘TO’ school), the neighborhood walkability model explained variance slightly better than the NT model (e.g., McFadden R-squared: 21.6% vs. 20.7%, respectively). In the NT model, frequent walkers’ schools were significantly more likely to be located in NTs than less frequent walkers’ schools (odds ratio [OR] = 5.11, 95% confidence interval [CI] = 1.12, 23.26, *P* = 0.035). The levels of neighborhood connection in everyday walkers and frequent walkers were significantly higher than those of less frequent walkers (OR = 1.54, 95% CI = 1.07, 2.21, *P* = 0.020; OR = 1.75, 95% CI = 1.17, 2.61, *P* = 0.007, respectively). The other odds ratios between the NT and neighborhood walkability models showed similar patterns.

**Table 3:** The results of multinomial logistic regression analyses for walking ‘TO’ school

	New Town Model						Neighborhood Walkability Model					
	Everyday walkers (*n* = 1227)		Frequent walkers (*n* = 178)		Everyday walkers vs frequent walkers		Everyday walkers (n = 1227)		Frequent walkers (n = 178)		Everyday walkers vs frequent walkers	
	OR (95% CI)	*P*	OR (95% CI)	*P*	OR (95% CI)	*P*	OR (95% CI)	*P*	OR (95% CI)	*P*	OR (95% CI)	*P*
School in NT (ref: no)												
Yes	3.28(0.76, 14.23)	0.113	5.11(1.12, 23.26)	**0.035**	1.56(0.91, 2.68)	0.109						
Neighborhood walkability												
Walking facilities							0.93(0.59, 1.46)	0.739	0.86(0.52, 1.43)	0.557	0.93(0.68, 1.27)	0.644
Aesthetics							1.27(0.76, 2.12)	0.360	1.45(0.82, 2.57)	0.196	1.15(0.81, 1.62)	0.446
Traffic safety							1.09(0.53, 2.28)	0.810	1.37(0.61, 3.10)	0.443	1.26(0.74, 2.12)	0.394
Crime safety							2.42(1.54, 3.80)	**< 0.001**	1.72(1.04, 2.83)	**0.035**	0.71(0.51, 0.98)	**0.038**
Social environments												
Neighborhood cohesion	0.84(0.45, 1.56)	0.573	0.95(0.49, 1.88)	0.890	1.14(0.73, 1.78)	0.568	0.89(0.47, 1.69)	0.721	0.97(0.49, 1.94)	0.933	1.09(0.69, 1.72)	0.708
Neighborhood connection	1.54(1.07, 2.21)	**0.020**	1.75(1.17, 2.61)	**0.007**	1.13(0.87, 1.48)	0.353	1.19(0.82, 1.74)	0.366	1.35(0.89, 2.05)	0.165	1.13(0.86, 1.49)	0.373
Safety volunteer (ref: No)												
Not sure	0.65(0.20, 2.11)	0.474	0.60(0.16, 2.23)	0.445	0.92(0.38, 2.25)	0.858	0.68(0.20, 2.24)	0.522	0.63(0.17, 2.37)	0.492	0.93(0.38, 2.27)	0.870
Yes	0.68(0.31, 1.48)	0.326	0.62(0.27, 1.45)	0.269	0.92(0.54, 1.55)	0.742	0.68(0.31, 1.49)	0.332	0.64(0.27, 1.51)	0.304	0.94(0.56, 1.60)	0.826
CCTV on school way (ref: No)												
Not sure	1.00(0.57, 1.73)	0.986	0.76(0.41, 1.42)	0.386	0.76(0.50, 1.16)	0.201	0.91(0.51, 1.60)	0.734	0.71(0.37, 1.33)	0.281	0.78(0.51, 1.18)	0.239
Yes	1.54(0.70, 3.36)	0.282	1.70(0.74, 3.92)	0.210	1.11(0.70, 1.77)	0.660	1.42(0.64, 3.14)	0.388	1.56(0.67, 3.64)	0.306	1.10(0.69, 1.76)	0.698
Walking over 100 m alone (ref: No)												
Not sure	1.25(0.39, 3.98)	0.709	1.64(0.46, 5.88)	0.451	1.31(0.61, 2.82)	0.487	1.20(0.37, 3.88)	0.760	1.56(0.43, 5.65)	0.502	1.30(0.60, 2.79)	0.507
Yes	0.66(0.39, 1.10)	0.106	1.22(0.69, 2.17)	0.492	1.87(1.29, 2.71)	**< 0.001**	0.64(0.38, 1.09)	0.101	1.17(0.65, 2.10)	0.607	1.82(1.25, 2.64)	**0.002**
Parent-perceived influence of COVID-19 (ref: No)												
Not sure	1.68(0.27, 10.51)	0.582	1.16(0.17, 8.16)	0.882	0.69(0.17, 2.79)	0.605	1.65(0.27, 10.05)	0.585	1.01(0.15, 6.94)	0.993	0.61(0.15, 2.53)	0.495
Yes	0.22(0.10, 0.47)	**< 0.001**	0.61(0.27, 1.37)	0.231	2.77(1.57, 4.91)	**< 0.001**	0.27(0.12, 0.61)	**0.001**	0.69(0.30, 1.62)	0.397	2.54(1.42, 4.57)	**0.002**
R-Squared												
Cox & Snell	0.216						0.224					
Nagelkerke	0.313						0.324					
McFadden	0.207						0.216					

Adjusted for distance to school, population density, grade, sex, and number of cars owned

Odds ratios were calculated relative to “less frequent walkers” (*n* = 92), except for everyday walkers (reference group) versus frequent walkers

CCTV, closed-circuit television; CI, confidence interval; COVID-19, coronavirus disease 2019; NT, new town; OR, odds ratio

**Table 4:** The results of multinomial logistic regression analyses for walking ‘FROM’ school

	New Town Model						Neighborhood Walkability Model					
	Everyday walkers (*n* = 1196)		Frequent walkers (*n* = 184)		Everyday walkers vs frequent walkers		Everyday walkers (n = 1196)		Frequent walkers (n = 184)		Everyday walkers vs frequent walkers	
	OR (95% CI)	*P*	OR (95% CI)	*P*	OR (95% CI)	*P*	OR (95% CI)	*P*	OR (95% CI)	*P*	OR (95% CI)	*P*
School in NT (ref: no)												
Yes	0.63(0.30, 1.30)	0.212	0.80(0.35, 1.82)	0.593	1.27(0.73, 2.23)	0.399						
Neighborhood walkability												
Walking facilities							0.94(0.62, 1.43)	0.779	0.85(0.53, 1.36)	0.506	0.91(0.67, 1.23)	0.530
Aesthetics							0.92(0.58, 1.45)	0.704	0.90(0.54, 1.50)	0.679	0.98(0.70, 1.38)	0.911
Traffic safety							1.52(0.76, 3.04)	0.232	2.44(1.13, 5.26)	**0.023**	1.60(0.96, 2.68)	0.074
Crime safety							1.43(0.94, 2.16)	0.094	1.23(0.77, 1.95)	0.382	0.86(0.63, 1.18)	0.359
Social environmental factors												
Neighborhood cohesion	1.45(0.81, 2.59)	0.213	2.17(1.15, 4.09)	**0.017**	1.50(0.96, 2.33)	0.075	1.46(0.81, 2.62)	0.209	2.17(1.14, 4.14)	**0.018**	1.49(0.95, 2.34)	0.082
Neighborhood connection	1.17(0.84, 1.63)	0.357	1.15(0.80, 1.67)	0.448	0.99(0.76, 1.28)	0.921	1.07(0.76, 1.50)	0.719	1.03(0.70, 1.51)	0.888	0.97(0.74, 1.26)	0.794
Safety volunteer (ref: No)												
Not sure	0.47(0.22, 1.01)	0.051	0.61(0.26, 1.42)	0.248	1.29(0.72, 2.33)	0.395	0.46(0.21, 0.99)	**0.046**	0.59(0.25, 1.38)	0.222	1.28(0.71, 2.32)	0.411
Yes	0.57(0.33, 1.00)	**0.050**	0.63(0.34, 1.16)	0.135	1.09(0.74, 1.62)	0.655	0.56(0.32, 0.99)	**0.048**	0.62(0.33, 1.15)	0.126	1.09(0.73, 1.62)	0.671
CCTV on school way (ref: No)												
Not sure	1.60(0.95, 2.71)	0.077	1.13(0.63, 2.03)	0.684	0.70(0.47, 1.06)	0.090	1.55(0.91, 2.64)	0.105	1.08(0.60, 1.96)	0.791	0.70(0.47, 1.05)	0.084
Yes	2.81(1.32, 6.01)	**0.008**	2.09(0.93, 4.70)	0.075	0.74(0.46, 1.19)	0.215	2.82(1.31, 6.08)	**0.008**	2.07(0.91, 4.71)	0.083	0.74(0.46, 1.18)	0.203
Walking over 100 m alone (ref: No)												
Not sure	1.08(0.41, 2.84)	0.871	1.01(0.32, 3.18)	0.990	0.93(0.40, 2.15)	0.865	1.13(0.43, 2.98)	0.802	1.07(0.34, 3.40)	0.910	0.94(0.41, 2.18)	0.893
Yes	0.97(0.60, 1.56)	0.896	1.81(1.07, 3.07)	**0.028**	1.87(1.30, 2.69)	**< 0.001**	1.04(0.64, 1.68)	0.888	1.96(1.15, 3.35)	**0.013**	1.90(1.31, 2.74)	**< 0.001**
Parent-perceived influence of COVID-19 (ref: No)												
Not sure	2.67(0.40, 17.95)	0.311	1.86(0.27, 12.64)	0.528	0.69(0.16, 3.05)	0.628	2.49(0.38, 16.31)	0.340	1.62(0.24, 10.84)	0.622	0.65(0.15, 2.90)	0.57
Yes	0.28(0.13, 0.59)	**< 0.001**	0.60(0.27, 1.34)	0.212	2.15(1.20, 3.88)	**0.011**	0.32(0.15, 0.69)	**0.004**	0.68(0.30, 1.57)	0.369	2.16(1.19, 3.92)	**0.012**
After-school activity	0.61(0.52, 0.71)	**< 0.001**	0.86(0.73, 1.01)	0.073	1.41(1.24, 1.61)	**< 0.001**	0.61(0.52, 0.71)	**< 0.001**	0.86(0.73, 1.02)	0.081	1.42(1.25, 1.62)	**< 0.001**
R-Squared												
Cox & Snell	0.244						0.249					
Nagelkerke	0.340						0.346					
McFadden	0.220						0.225					

Adjusted for distance to school, population density, grade, sex, and number of cars owned

Odds ratios were calculated relative to “less frequent walkers” (*n* = 117), except for everyday walkers (reference group) versus frequent walkers

CCTV, closed-circuit television; CI, confidence interval; COVID-19, coronavirus disease 2019; NT, new town; OR, odds ratio

In the neighborhood walkability model (Table 3), the levels of neighborhood crime safety in everyday walkers and frequent walkers were significantly higher than those in less frequent walkers (OR = 2.42, 95% CI = 1.54, 3.80, *P* < 0.001; OR = 1.72, 95% CI = 1.04, 2.83, *P* = 0.035, respectively). When comparing frequent walkers with everyday walkers (reference group), significantly lower odds were observed for neighborhood crime safety (OR = 0.71, 95% CI = 0.51, 0.98, *P* = 0.038). Frequent walkers tended to walk more than 100 m alone compared to everyday walkers (OR = 1.82, 95% CI = 1.25, 2.64, *P* = 0.002). In both the NT and neighborhood walkability models, children of parents who perceived a larger influence of COVID-19 tended to walk less frequently to school.

Despite marginal increases in McFadden R-squared values, the walking ‘FROM’ school models (Table 4) explained more variance than the walking ‘TO’ school models (Table 3) in both the NT and neighborhood walkability models (difference: 0.013 and 0.009, respectively). Except the school in NT and neighborhood walkability variables, all ORs in the NT and neighborhood walkability models showed similar patterns. In the neighborhood walkability model, the level of neighborhood traffic safety in frequent walkers was significantly higher than that of less frequent walkers (OR = 2.44, 95% CI = 1.13, 5.26, *P* = 0.023). Similarly, the level of neighborhood cohesion was significantly higher in frequent walkers than in less frequent walkers (OR = 2.17, 95% CI = 1.14, 4.14, *P* = 0.018). Regarding the safety measures, in both NT and neighborhood walkability models, everyday walkers were less likely to report safety volunteers compared to less frequent walkers (both models: OR = 0.56, 95% CI = 0.32, 0.99, *P* = 0.048). However, everyday walkers were more likely to report CCTVs along children’s travel routes (OR = 2.82, 95% CI = 1.31, 6.08, *P* = 0.008). Similarly, higher odds of walking more than 100 m alone were observed in frequent walkers than in everyday walkers (OR = 1.90, 95% CI = 1.31, 2.74, *P* < 0.001). The same trend as walking ‘TO’ school was observed in terms of parent-perceived influence of COVID-19, in which everyday walkers were impacted the least on their school travel mode. The frequency of after-school activities was also negatively associated with walking from school.

## Discussion

This study examined the associations of children’s neighborhood built and social environments and safety with their walking to and from school. Furthermore, we explored these associations by neighborhood urban planning and design (NT) and neighborhood walkability. We found that crime safety, neighborhood connection, and school in an NT were positively associated with the frequency of walking to school. Walking from school had positive associations with traffic safety, neighborhood cohesion, and CCTVs, but negative associations with safety volunteers and after-school activities. Children who walked more than 100 m alone and those whose parents perceived a larger influence of COVID-19 tended to walk less frequently to and from school.

The current study showed that the percentage of children walking to and from school every day was 82% (more than half a month: 94%) and 80% (92%), respectively, which is extremely high compared to other developed countries [24, 41]. However, recent social changes in Japan may hinder the high prevalence of walking to and from school. For example, the number of public elementary schools in Japan have decreased, particularly in rural areas, due to school consolidations, which has resulted in a gradual increase in the number of school buses [42]. Another example of social change is a rapid rise in after-school activities, such as cram schools, owing to the growing parental enthusiasm for education as well as double-income households [43, 44]. There may be cases where parents pick up children straight after-school and drive them to activities, which potentially reduces the chance of children walking back from school. These social changes may also reduce opportunities and time for leisure PA, such as playing in parks [3]. To tackle these issues and help children meet their PA recommendations, it is important to consider promoting walking to and from school and overall PA through different domains, namely transportation, education, leisure, and household [1]. For instance, active and safe environments for children can be created by positioning bus stops away from school gates to allow children to gain some walking distance and by providing sufficient physical education and active lessons in schools [45, 46].

As we hypothesized, NTs had significantly higher perceived neighborhood walkability (i.e., walking facilities, aesthetics, traffic safety, and crime safety) than non-NTs. The walkable and safe features of NTs, as well as their highly connected greenways, may support walking to school [15, 47, 48]. After 50 years of constructing NTs, they have been renewed since around 2000, and it is necessary for urban planners to conserve the environment of NTs that support walking to and from school by securing walkable and safe neighborhood built environments [49].

Crime safety, a subscale of walkability, was positively associated with the frequency of walking to school, and the existence of a road section where children walked alone had a negative association with the frequency of walking to and from school. Children walking on lone road sections may have increased as a consequence of the declining birthrate and different timings and travel modes from other children, including siblings and friends, especially when going home (partially due to after-school activities) [22]. Thus, measures to reduce parental fear of crime and road sections where children walk alone are required to encourage walking to and from school. For instance, increasing “natural surveillance” or “eyes on the street” by local residents and watching over streets by volunteers may be necessary in the short term [50, 51]. In the long term, housing design that enables residents to view the street (e.g., installing front windows and see-through exterior walls) can be used to further improve the safety of neighborhood environments. Surprisingly, the presence of a safety volunteer was negatively associated with the frequency of walking from school. Safety volunteers tend to be arranged in areas where traditional communities exist, such as rural areas. Such areas are less densely populated and have fewer schools, which leads to fewer eyes on the street and a longer distance to school [52]. Consequently, children are generally less likely to walk to or from school. In this study, we included population density and distance to school in the analyses to account for urban and rural areas; however, there might be spurious correlations with other factors in relation to rural areas, such as the complexity of travel other than to/from school [53]. Moreover, traditional safety volunteering, such as patrolling the neighborhood or supervising road crossings, may no longer be possible for the new generation. Safety volunteering, which is generally physically demanding and time-intensive, has become a burdensome activity for parents and older residents due to the increase in double-income households and aging, thus reducing the number of volunteers [22]. To make up for the reduced number of traditional volunteers, ordinary residents should watch out for children walking to and from school during their daily activities such as dog walking and jogging in the neighborhood [20, 22]. Alternatively, the presence of CCTVs was positively associated with the frequency of walking from school. It is likely that CCTVs give parents a sense of security. Given after-school activities, 24-h surveillance may be necessary to respond to social changes, especially at the times of children’s travel from school.

The safety measures noted above are inseparably related to social environments [18]. Although direct relations between the safety measures and social environments were not investigated in the current study, neighborhood connection and cohesion were positively associated with walking to and from school, which is consistent with previous studies [18, 37]. Contrary to the traditional safety volunteering approach described in the previous paragraph, safety volunteering integrated into daily community activities may engage more local residents, which can boost and enrich the neighborhood social environment or social capital [54, 55]. There might be a mediating path wherein safety volunteers boosted neighborhood social environments, which in turn supported walking to and from school. However, it should be noted that the presence of CCTVs might be accepted as a sign of a lack of social capital to carry out residents’ activities for safety and underestimate their importance, which can accelerate the loss of social cohesion [56].

Schools face a number of challenges in managing a safe school run effectively during the COVID-19 pandemic. The Japanese government provided emergency statutory guidance for schools nationwide to advise children to avoid contact with others and maintain physical distance from others when traveling to and from school and to ensure their safety from crime in anticipation of an increasing number of children walking alone [57]. The current study demonstrated that children of parents who perceived a larger influence of COVID-19 had a smaller chance of being categorized into ‘everyday walker’ than ‘frequent walker’ or ‘less frequent walker,’ indicating that some parents might instead drive their children to and from school because of their concerns about the risk of both infection and crime. A situation where more parents worked from home might influence changes in children’s travel mode to and from school during the COVID-19 pandemic.

The strengths of this study are its use of a relatively large sample from a mix of urban, suburban, and rural areas, focusing on the differences in built and social environments, and employing various safety measures in relation to children’s walking to and from school. We also integrated subjective and objective data derived from participant surveys and geographic information systems. Despite these strengths, our study has some limitations. First, this study was cross-sectional. Hence, we were unable to draw causal inferences on the relations between the neighborhood environment and walking to and from school. Second, only the built environment around the children’s homes were assessed, not around their schools [14]. Third, the participants included in the current study may not be representative for parents in Chiba prefecture as the participants were those who voluntarily registered with a research company. Fourth, the current findings may not be generalizable to other parts of Japan, as the data were collected from a specific geographical region of Chiba prefecture. Future research should consider a longitudinal survey with a large-scale sample size across Japan, which will investigate how changes in the neighborhood environment influence children’s travel mode. Japan has maintained a high prevalence of children walking to and from school even after motorization during the 1960s–80s. However, given the rapid social changes over the past few decades, new approaches to overcome sectionalism, such as collaboration with different stakeholders from education, public health, and urban planning will be required to support this behavior over time [58]. Globally, inter-sectoral collaboration would serve as good references. For instance, the Safe Routes to School program has developed a collaborative relationship between school and local planners, and the Active Living by Design program has highlighted cross-sector collaboration and community partnerships [59, 60].

## Conclusion

This study examined the associations of children’s neighborhood built and social environments, and safety with children walking to and from schools in Chiba, Japan. Consistent with previous research, we found that four-fifths of children walked to and from school every day. Walking to school was positively associated with crime safety, neighborhood connections, and school in NTs. Walking from school had positive associations with traffic safety, neighborhood cohesion, and CCTVs, but negative associations with safety volunteers and after-school activities. Children who walked more than 100 m alone and those whose parents perceived a larger influence of COVID-19 tended to walk less frequently to and from school. These findings imply that recent social changes such as declining birthrate, decline in public elementary schools, and increasing after-school activities may change parental attitudes toward children’s walking to and from school, and subsequently their mode of school travel over time. To maintain the high prevalence of walking to and from school in Japan, multidisciplinary approaches involving different stakeholders from education, public health, and urban planning are required to overcome sectionalism and support this behavior in the long term.

## Supplementary Information

**Additional file 1.** STROBE statement checklist of items for cross-sectional studies.

## Data Availability

The datasets used and analyzed during the current study are available from the corresponding author on reasonable request.

## Abbreviations

ANEWS: Abbreviated Neighborhood Environment Walkability Scale
CCTV: closed-circuit television
CI: confidence interval
COVID-19: coronavirus disease 2019
NEWS-Y: Neighborhood Environment Walkability Scale in Youth
NT: new town
OR: odds ratio
PA: physical activity
SD: standard deviation
